# The *TP53* Arg72Pro and *MDM2* 309G>T polymorphisms are not associated with breast cancer risk in *BRCA1* and *BRCA2* mutation carriers

**DOI:** 10.1038/sj.bjc.6605279

**Published:** 2009-08-25

**Authors:** O M Sinilnikova, A C Antoniou, J Simard, S Healey, M Léoné, D Sinnett, A B Spurdle, J Beesley, X Chen, kConFab, M H Greene, J T Loud, F Lejbkowicz, G Rennert, S Dishon, I L Andrulis, OCGN, S M Domchek, K L Nathanson, S Manoukian, P Radice, I Konstantopoulou, I Blanco, A L Laborde, M Durán, A Osorio, J Benitez, U Hamann, F B L Hogervorst, T A M van Os, H J P Gille, HEBON, S Peock, M Cook, C Luccarini, D G Evans, F Lalloo, R Eeles, G Pichert, R Davidson, T Cole, J Cook, J Paterson, C Brewer, EMBRACE, D J Hughes, I Coupier, S Giraud, F Coulet, C Colas, F Soubrier, E Rouleau, I Bièche, R Lidereau, L Demange, C Nogues, H T Lynch, GEMO,,, R K Schmutzler, B Versmold, C Engel, A Meindl, N Arnold, C Sutter, H Deissler, D Schaefer, U G Froster, GC-HBOC,,,,,,,, K Aittomäki, H Nevanlinna, L McGuffog, D F Easton, G Chenevix-Trench, D Stoppa-Lyonnet

**Affiliations:** 1 Unité Mixte de Génétique Constitutionnelle des Cancers Fréquents, Hospices Civils de Lyon / Centre Léon Bérard, Lyon 69373, France; 2 Laboratoire de Génétique Moléculaire, Signalisation et Cancer, UMR5201 CNRS, Université de Lyon, Lyon 69373, France; 3 Cancer Research UK, Genetic Epidemiology Unit, Department of Public Health and Primary Care, University of Cambridge, Cambridge CB1 8RN, UK; 4 Canada Research Chair in Oncogenetics, Cancer Genomics Laboratory, Centre Hospitalier Universitaire de Quebec and Laval University, Quebec, Canada G1V 4G2; 5 Queensland Institute of Medical Research, Brisbane QLD 4029, Australia; 6 Division of Hematology-Oncology, Research Center, CHU Sainte-Justine, Montreal, Canada H3T 1C5; 7 Department of Pediatrics, University of Montreal, Montreal, Canada H3T 1C5; 8 Peter MacCallum Cancer Center, Melbourne, VIC 3002, Australia; 9 Clinical Genetics Branch, Division of Cancer Epidemiology and Genetics, National Cancer Institute, Rockville, MD 20852-7231, USA; 10 CHS National Cancer Control Center and Department of Community Medicine and Epidemiology, Carmel Medical Center and Technion Faculty of Medicine, Haifa 34362, Israel; 11 Ontario Cancer Genetics Network, Cancer Care Ontario, Department of Molecular Genetics, University of Toronto, Toronto, ON, Canada M5G 1X5; 12 Samuel Lunenfeld Research Institute, Mount Sinai Hospital, Toronto, ON, Canada M5G 1X5; 13 Department of Medicine, Abramson Cancer Center, University of Pennsylvania School of Medicine, Philadelphia, PA 19104, USA; 14 Unit of Medical Genetics, Fondazione IRCCS Istituto Nazionale Tumori, Milano, Italy; 15 Department of Experimental Oncology, Fondazione IRCCS Istituto Nazionale Tumori, Milano 20133, Italy; 16 IFOM, Fondazione Istituto FIRC di Oncologia Molecolare, Milano 20139, Italy; 17 Molecular Diagnostics Laboratory, IRRP, NCSR ‘Demokritos’, 15310 Ag. Paraskevi, Athens, Greece; 18 Cancer Genetic Counseling Program, Institut Català d'Oncologia, Barcelona 08907, Spain; 19 Servicio de Genética, Hospital de la Santa Creu i Sant Pau, Barcelona 08025, Spain; 20 Laboratorio de Genética del Cáncer, Instituto de Biología y Genética Molecular, Universidad de Valladolid, Valladolid 47002, Spain; 21 Human Cancer Genetics Programme, Spanish National Cancer Research Centre (CNIO), Madrid 28029, Spain; 22 Deutsches Krebsforschungszentrum (DKFZ), Molecular Genetics of Breast Cancer, Heidelberg 69120, Germany; 23 Department of Pathology and the Family Cancer Clinic, Netherlands Cancer Institute, Amsterdam 1066 CX, The Netherlands; 24 Department of Clinical Genetics, Amsterdam University Medical Centre, Amsterdam 1105 AZ, The Netherlands; 25 Department of Clinical Molecular Genetics, Free University Medical Centre, Amsterdam 1081 HV, The Netherlands; 26 Department of Oncology, University of Cambridge, Cambridge CB1 8RN, UK; 27 Manchester Academic Health Sciences Centre, Central Manchester University Hospitals NHS Foundation Trust, Manchester M13 OJH, UK; 28 Translational Cancer Genetics Team, The Institute of Cancer Research and Royal Marsden NHS Foundation Trust, Sutton, Surrey SM2 5NG, UK; 29 Clinical Genetics, Guy's Hospital, London SE1 9RT, UK; 30 Ferguson-Smith Centre for Clinical Genetics, Glasgow G3 8SJ, UK; 31 West Midlands Regional Genetics Service, Birmingham Women's Hospital Healthcare NHS Trust, Edgbaston, Birmingham B15 2TG, UK; 32 Sheffield Clinical Genetics Service, Sheffield Children's Hospital, Sheffield S10 2TH, UK; 33 Department of Clinical Genetics, East Anglian Regional Genetics Service, Addenbrookes Hospital, Cambridge CB2 8QE, UK; 34 Department of Clinical Genetics, Royal Devon & Exeter Hospital, Exeter EX2 5DW, UK; 35 Department of Clinical Medicine, Trinity College Centre for Health Sciences, Adelaide & Meath Hospital, Dublin 24, Ireland; 36 Service de Génétique Médicale, Unité d'Oncogénétique, CHU Arnaud de Villeneuve, Montpellier 34295, France; 37 Unité d'Oncogénétique, CRLCC Val d'Aurelle, Montpellier 34295, France; 38 Groupe Hospitalier Pitié-Salpêtriére, Assistance Publique-Hopitaux de Paris, Laboratoire d'Oncogénétique et Angiogénétique Moléculaire, Université Pierre et Marie Curie, Paris 75651, France; 39 INSERM U735 / Oncogenetics, Centre René Huguenin, Saint-Cloud 92210, France; 40 Epidémiologie Clinique, Oncogénétique, Centre René Huguenin, Saint-Cloud 92210, France; 41 Department of Preventive Medicine, Creighton University, Omaha, NE 68178, USA; 42 INSERM U509, Service de Génétique Oncologique, Institut Curie, Université Paris-Descartes, Paris 75248, France; 43 Centre for hereditary Breast and Ovarian Cancer, Department of Obstetrics and Gynaecology, University of Cologne, Cologne 50924, Germany; 44 Institute for Medical Informatics, Statistics and Epidemiology, University of Leipzig, Leipzig 04103, Germany; 45 Department of Obstetrics and Gynaecology, Technical University Munich, Munich 80333, Germany; 46 Department of Obstetrics and Gynaecology, University of Schleswig-Holstein, Kiel 24105, Germany; 47 Institute of Human Genetics, University of Heidelberg, Heidelberg 69117, Germany; 48 Department of Obstetrics and Gynaecology, University of Ulm, Ulm 89069, Germany; 49 Institute of Human Genetics, University of Frankfurt, Frankfurt 60325, Germany; 50 Institute of Human Genetics, University of Leipzig, Leipzig 04103, Germany; 51 Department of Clinical Genetics, Helsinki University Central Hospital, Helsinki, 00029 HUS, Finland; 52 Department of Obstetrics and Gynecology, Helsinki University Central Hospital, Helsinki, 00029 HUS, Finland

**Keywords:** *TP53*, *MDM2*, *BRCA1/2*, breast cancer, polymorphism, risk

## Abstract

**Background:**

The *TP53* pathway, in which *TP53* and its negative regulator *MDM2* are the central elements, has an important role in carcinogenesis, particularly in *BRCA1-* and *BRCA2-*mediated carcinogenesis. A single nucleotide polymorphism (SNP) in the promoter region of *MDM2* (309T>G, rs2279744) and a coding SNP of *TP53* (Arg72Pro, rs1042522) have been shown to be of functional significance.

**Methods:**

To investigate whether these SNPs modify breast cancer risk for *BRCA1* and *BRCA2* mutation carriers, we pooled genotype data on the *TP53* Arg72Pro SNP in 7011 mutation carriers and on the *MDM2* 309T>G SNP in 2222 mutation carriers from the Consortium of Investigators of Modifiers of *BRCA1/2* (CIMBA). Data were analysed using a Cox proportional hazards model within a retrospective likelihood framework.

**Results:**

No association was found between these SNPs and breast cancer risk for *BRCA1* (*TP53*: per-allele hazard ratio (HR)=1.01, 95% confidence interval (CI): 0.93–1.10, *P*_trend_=0.77; *MDM2*: HR=0.96, 95%CI: 0.84–1.09, *P*_trend_=0.54) or for *BRCA2* mutation carriers (*TP53*: HR=0.99, 95%CI: 0.87–1.12, *P*_trend_=0.83; *MDM2*: HR=0.98, 95%CI: 0.80–1.21, *P*_trend_=0.88). We also evaluated the potential combined effects of both SNPs on breast cancer risk, however, none of their combined genotypes showed any evidence of association.

**Conclusion:**

There was no evidence that *TP53* Arg72Pro or *MDM2* 309T>G, either singly or in combination, influence breast cancer risk in *BRCA1* or *BRCA2* mutation carriers.

The *TP53* pathway is crucial for tumour suppression, acting through regulation of cell-cycle control, apoptosis, senescence and DNA repair. The *TP53* gene and its negative regulator *MDM2* are central to this pathway, promoting polyubiquitination and degradation of TP53, and also controlling the TP53 synthesis (Toledo and Wahl, 2006; Candeias *et al*, 2008). Inactivation of the *TP53* pathway has an important role in *BRCA1*- and *BRCA2*-associated tumourigenesis. *BRCA1* and *BRCA2* mutations are associated with genomic instability caused by defective cell-cycle checkpoint and DNA damage repair (Deng, 2006). Mouse model studies have highlighted functional links between these genes. Biallelic inactivation of *BRCA1* and *BRCA2* in mice have shown that embryonic lethality because of growth retardation can be partially rescued in a *Trp53* null background (Evers and Jonkers, 2006). The development of mammary tumours in conditional *BRCA1* and *BRCA2* knockout mice was considerably accelerated in a *Trp53* knockout background (Evers and Jonkers, 2006). In addition, a high incidence of *TP53* mutations has been found in breast tumours of human *BRCA1* and *BRCA2* mutation carriers (Greenblatt *et al*, 2001; Manie *et al*, 2009). The observed interactions between *TP53* and *BRCA* pathways are integral to the progression of tumourigenesis in breast cancer.

A *TP53* polymorphism (rs1042522) has been found to be of functional significance, with the Pro72 allele being less efficient than Arg72 at inducing apoptosis, mainly due to weaker binding and ubiquitination by MDM2 of the Pro72 variant protein (Dumont *et al*, 2003; Osorio *et al*, 2006). An SNP in the promoter region of *MDM2* (309T>G, rs2279744) has been shown to increase *MDM2* transcriptional activity, thus attenuating the *TP53* pathway (Bond *et al*, 2004). This latter SNP was associated with an earlier onset of breast cancer in Li–Fraumeni patients carrying *TP53* mutations (Bougeard *et al*, 2006; Ruijs *et al*, 2007). The effect on breast cancer risk of the *TP53* Arg72Pro and the *MDM2* 309T>G polymorphisms, separately and in combination, was investigated in a large case–control study by the Breast Cancer Association Consortium (BCAC), but no association was detected (Schmidt *et al*, 2007). However, several smaller studies examined these polymorphisms in *BRCA1* and *BRCA2* mutation carriers (Martin *et al*, 2003; Tommiska *et al*, 2005; Copson *et al*, 2006; Osorio *et al*, 2006; Wasielewski *et al*, 2007; Yarden *et al*, 2008), and some suggested an association between the *TP53* Pro72 and the *MDM2* 309G alleles with an earlier age at breast cancer diagnosis (Martin *et al*, 2003; Tommiska *et al*, 2005; Osorio *et al*, 2006; Yarden *et al*, 2008). We therefore investigated the associations between breast cancer risk and these *TP53* and *MDM2* polymorphisms in a large series of *BRCA1* and *BRCA2* mutation carriers from the Consortium of Investigators of Modifiers of *BRCA1*/*2* (CIMBA) (Chenevix-Trench *et al*, 2007).

## Materials and methods

### Study sample

Eligibility was restricted to female carriers with pathogenic mutations in *BRCA1* or *BRCA2* who were ⩾18 years. Data were obtained from 13 CIMBA studies (Table 1). The majority of carriers were recruited through cancer genetics clinics offering genetic testing, and enrolled into national or regional studies. Information collected included the year of birth; mutation description; age at last followup; ages at breast and ovarian cancer diagnosis; and age at bilateral prophylactic mastectomy. Information was also available on the country of residence, which was defined to be the country of the clinic at which the carrier family was recruited for the study. Related individuals were identified through a unique family identifier. Further details of the information collected on the *BRCA1* and *BRCA2* mutation carriers and other details of the CIMBA initiative can be found elsewhere. Additional specific acknowledgements to the CIMBA collaborating centres are included in the Supplementary Appendix. (http://www.srl.cam.ac.uk/consortia/cimba/index.html) (Chenevix-Trench *et al*, 2007). All carriers participated in clinical and research studies at the host institutions under IRB-approved protocols.

### Genotyping

We pooled genotype data from studies within CIMBA that had previously genotyped polymorphisms rs1042522 and rs2279744 (see Table 1). Deviation from Hardy–Weinberg equilibrium among unrelated subjects was evaluated separately for each SNP and study. There was evidence for deviation for only one study (*P*=0.03), but cluster plot examination did not show any unusual pattern and the study was included in the analysis. Where available study specific genotyping quality control data were examined and data were included if the call rate was over 95% and the concordance among duplicates was over 98%.

### Statistical analysis

Mutation carriers were classified according to their age at diagnosis of breast cancer or their age at last follow up. For this purpose, individuals were censored at the age of first breast cancer diagnosis, ovarian cancer diagnosis, bilateral prophylactic mastectomy or the age at last observation. Only individuals censored at breast cancer diagnosis were assumed to be affected (Table 2).

To correct for a potential bias related to the fact that *BRCA1* and *BRCA2* mutation carriers are not randomly sampled with respect to their disease status, the data were analysed within a survival analysis framework, by modelling the retrospective likelihood of the observed genotypes conditional on the disease phenotypes. A detailed description of the retrospective likelihood approach has been published (Antoniou *et al*, 2007). We used a Cox proportional hazards model, where the effect of each SNP was modelled either as a per-allele hazard ratio (HR) or using separate HRs for heterozygotes and homozygotes. To assess the combined effects of the SNPs, we fitted a model in which a separate HR parameter was estimated for each multilocus genotype. More details of the statistical analysis can be found elsewhere (Antoniou *et al*, 2008).

## Results

In total, 7011 *BRCA1* and *BRCA2* mutation carriers were genotyped for *TP53* Arg72Pro and 2222 mutation carriers were genotyped for *MDM2* 309T>G (Table 1). Table 2 shows summary statistics for the cohort of *BRCA1* and *BRCA2* mutation carriers with an observed genotype for either the *TP53* or *MDM2* polymorphism. There was no evidence of an association between either SNP and breast cancer risk in *BRCA1* or *BRCA2* mutation carriers combined or analysed separately (*TP53* Arg72Pro: *P*_trend_=0.89, 0.77 and 0.83, respectively; *MDM2* 309T>G: *P*_trend_=0.60, 0.54 and 0.88, respectively) (Table 3). There was no evidence for heterogeneity in the HRs between studies (*TP53* Arg72Pro: *P*=0.22 and 0.93, *MDM2* 309T>G: *P*=0.11 and 0.82 for *BRCA1* or *BRCA2* mutation carriers respectively). The HRs for the 9 *TP53–MDM2* combined genotypes, estimated separately in *BRCA1* and *BRCA2* mutation carriers, ranged between 0.72 and 1.31, but none of them were significant.

## Discussion

To our knowledge, this is the largest study to investigate the hypothesis that *TP53* Arg72Pro and *MDM2* 309T>G influence breast cancer risk in *BRCA1* and *BRCA2* mutation carriers individually or in combination. Our findings of no association for these SNPs suggest that they have little or no effect on *BRCA*-related breast cancer risk. These results are consistent with the absence of risk association in the recent *TP53* haplotype analysis, involving Arg72Pro and an intronic polymorphism c.97-147ins16 bp, in a series of 2932 *BRCA1* and *BRCA2* carriers from CIMBA (Osorio *et al*, 2008). Our sample of mutation carriers had power of approximately 75% for *TP53* and 40% for *MDM2* to detect significant associations (*P*<0.05) for a per-allele HR of 1.1 and power of 100 and 90% respectively for a HR of 1.2, suggesting that we can reliably dismiss previously suggested associations (Martin *et al*, 2003; Osorio *et al*, 2006; Yarden *et al*, 2008).

Yarden *et al* showed that the *MDM2* GG genotype among Ashkenazi *BRCA1/2* mutations carriers was significantly associated with breast cancer diagnosed <age 51 (*P*=0.019) (Yarden *et al*, 2008). However, we did not find any evidence of an increased risk for the GG homozygotes among the 217 carriers of the *BRCA1* Ashkenazi mutations 185delAG and 5382insC (HR=0.98, 95%CI 0.48–2.01) in this series.

The BCAC study of 5191 cases and 3834 controls found no evidence of an association of *TP53* Arg72Pro and *MDM2* 309T>G either with breast cancer overall or with oestrogen receptor (ER) status of tumours (Schmidt *et al*, 2007). As the majority of *BRCA1* mutation-associated breast tumours are ER-negative (Lakhani *et al*, 2005), the absence of an association in our study of breast cancer with the *TP53* and *MDM2* SNPs in *BRCA1* mutation carriers is consistent with the lack of an association with ER-negative cancers in the general population.

## Figures and Tables

**Table 1:** Number of *BRCA1* and *BRCA2* mutation carriers by study and by single nucleotide polymorphism (SNP)

**Study**	**Country**	***TP53* Arg72Pro (rs1042522)**	***MDM2* 309T>G (rs2279744)**	**Genotyping platform**
Spanish National Cancer Centre (CNIO)	Spain	788	0	Restriction enzyme digestion
Deutsches Krebsforschungszentrum (DKFZ)	Germany	170	0	PCR-based RFLP
Epidemiological study of *BRCA1* and *BRCA2* mutation carriers (EMBRACE)	U.K. and Eire	1131	0	iPLEX
Genetic Modifiers of cancer risk in *BRCA1/2* mutation carriers (GEMO)	France and U.S.A.	1405	1357	Taqman
German Consortium of Hereditary Breast and Ovarian Cancer (GC-HBOC)	Germany	815	0	Taqman
Helsinki Breast Cancer Study (HEBCS)	Finland	188	187	rs1042522: Amplifluor(tm) fluorescent genotyping (Kbiosciences); rs2279744: RFLP
HEreditary Breast and Ovarian study Netherlands (HEBON)	The Netherlands	438	432	Taqman
INterdisciplinary HEalth Research International Team BReast CAncer susceptibility (INHERIT BRCAs)	Quebec-Canada	146	155	Taqman
kConFab	Australia	790	0	iPLEX
National Cancer Institute (NCI)	USA	190	0	Taqman
National Israeli Cancer Control Center (NICCC)	Israel	470	0	Taqman
Ontario Cancer Genetics Network (OCGN)	Canada	84	91	Taqman
University of Pennsylvania (UPENN)	USA	396	0	iPLEX
Total		7011	2222	

**Table 2:** Summary characteristics for the 7109 eligible *BRCA1* and *BRCA2* carriers used in the analysis and typed for either single nucleotide polymorphism (SNP)

	***BRCA1***		***BRCA2***	
**Characteristic**	**Unaffected**	**Breast cancer**	**Unaffected**	**Breast cancer**
Number	2055	2567	1051	1436
Person-years follow-up	87 571	104 679	46 315	63 080
Median age at censure (IQR)	41 (33–51)	40 (34–46)	42 (34–52)	43 (37–50)
				
*Age at censure (years), N* (%)				
<30	327 (15.9)	225 (8.8)	139 (13.2)	78 (5.4)
30–39	584 (28.4)	1052 (41.0)	296 (28.1)	462 (32.2)
40–49	574 (27.9)	880 (34.3)	286 (27.2)	511 (35.6)
50–59	364 (17.7)	296 (11.5)	196 (18.7)	278 (19.4)
60–69	134 (6.5)	87 (3.4)	82 (7.8)	81 (5.6)
70+	72 (3.5)	27 (1.0)	52 (4.9)	26 (1.8)
				
*Year of birth, N* (%)				
<1920	18 (0.9)	32 (1.3)	12 (1.1)	10 (0.7)
1920–29	63 (3.1)	117 (4.6)	39 (3.7)	83 (5.8)
1930–39	171 (8.3)	267 (10.4)	96 (9.1)	196 (13.7)
1940–49	326 (15.9)	657 (25.6)	143 (13.6)	358 (24.9)
1950–59	481 (23.4)	820 (31.9)	241 (22.9)	459 (32.0)
1960+	996 (48.5)	674 (26.3)	520 (49.5)	330 (23.0)

Abbreviation: IQR=interquartile range.

**Table 3:** Genotype frequencies by mutant gene and breast cancer status with hazard ratio (HR) estimates

	**Unaffected (%)**	**Affected (%)**	**HR**	**95% CI**	***P*-value**
*TP53 Arg72Pro (rs1042522)*					
*BRCA1*/2					
GG	1660 (54.4)	2164 (54.7)	1.00		
GC	1178 (38.6)	1508 (38.1)	1.00	0.92–1.10	
CC	214 (7.0)	287 (7.3)	1.01	0.85–1.20	
2-df test					0.99
Per allele			1.01	0.94–1.08	0.89
					
*BRCA1*					
GG	1127 (56.0)	1399 (55.2)	1.00		
GC	748 (37.2)	947 (37.4)	1.01	0.90–1.13	
CC	138 (6.9)	188 (7.4)	1.03	0.84–1.27	
2-df test					0.96
Per allele			1.01	0.93–1.10	0.77
					
*BRCA2*					
GG	533 (51.3)	765 (53.7)	1.00		
GC	430 (41.4)	561 (39.4)	0.98	0.84–1.14	
CC	76 (7.3)	99 (6.9)	0.99	0.72–1.36	
2-df test					0.95
Per allele			0.99	0.87–1.12	0.83
					
*MDM2 309T*>*G (rs2279744)*					
*BRCA1/2*					
TT	358 (40.3)	530 (39.8)	1.00		
TG	405 (45.6)	615 (46.1)	0.99	0.84–1.18	
GG	126 (14.2)	188 (14.1)	0.93	0.73–1.17	
2-df test					0.79
Per allele			0.97	0.87–1.08	0.60
					
*BRCA1*					
TT	275 (39.7)	369 (39.5)	1.00		
TG	323 (46.6)	443 (47.4)	0.98	0.81–1.19	
GG	95 (13.7)	123 (13.2)	0.91	0.67–1.19	
2-df test					0.78
Per allele			0.96	0.84–1.09	0.54
					
*BRCA2*					
TT	83 (42.4)	161 (40.5)	1.00		
TG	82 (41.8)	172 (43.2)	1.07	0.77–1.50	
GG	31 (15.8)	65 (16.3)	0.93	0.60–1.44	
2-df test					0.83
Per allele			0.98	0.80–1.21	0.88
