# Supplementary material for: The TP53 Arg72Pro and MDM2 309G>T polymorphisms are not associated with breast cancer risk in BRCA1 and BRCA2 mutation carriers
Source: Br J Cancer. 2009 Aug 25;101(8):1456–60. doi: 10.1038/sj.bjc.6605279 (PMC2768437; doi:10.1038/sj.bjc.6605279)
Supplement: Supplementary Appendix [file 6605279x1.doc]

**SUPPLEMENTARY INFORMATION**

**Appendix: CIMBA Collaborating Centres Specific Acknowledgements**

Interdisciplinary Health Research International Team Breast Cancer susceptibility (INHERIT): Jacques Simard, Francine Durocher, Rachel Laframboise, Marie Plante, Centre Hospitalier Universitaire de Québec & Laval University, Québec, Canada ; Daniel Sinnett, Sainte-Justine Hospital & University of Montreal, Québec, Canada. Peter Bridge, Jilian Parboosingh, Molecular Diagnostic Laboratory, Alberta Children’s Hospital, Calgary, Canada; Jocelyne Chiquette, Hôpital du Saint-Sacrement, Québec, Canada ; Bernard Lespérance, Hôpital du Sacré-Cœur de Montréal, Montréal, Canada.

Collaborators of the DKFZ Study: Javeriana University, Bogota, Colombia: Diana Torres, Angela Beltran Lopez, Viviana Ariza, Angela Umana, Ignacio Bricenio; Clinicians: Jose F. Robledo, Jose J. Caicedo, Country Clinic, Bogota, Colombia; Shaukat Khanum Memorial Cancer Hospital & Research Centre, Lahore, Pakistan: Muhammad U. Rashid, Faisal Sultan.

The Hereditary Breast and Ovarian Cancer Research Group Netherlands (HEBON) Collaborating Centers: Coordinating center: Netherlands Cancer Institute, Amsterdam: Frans Hogervorst, Linde Braaf , Senno Verhoef, Anouk Pijpe, Laura van ‘t Veer, Flora van Leeuwen, Matti Rookus; Erasmus Medical Center, Rotterdam: Margriet Collée, Ans van den Ouweland, Mieke Kriege, Mieke Schutte, Maartje Hooning, Caroline Seynaeve; Leiden University Medical Center, Leiden: Rob Tollenaar, Christi van Asperen, Juul Wijnen, Maaike Vreeswijk, Peter Devilee; Radboud University Nijmegen Medical Center, Nijmegen: Nicoline Hoogerbrugge, Marjolijn Ligtenberg; University Medical Center Utrecht, Utrecht: Margreet Ausems, Rob van der Luijt; Amsterdam Medical Center: Cora Aalfs, Theo van Os; VU University Medical Center, Amsterdam: Hanne Meijers-Heijboer, Hans Gille; University Hospital Maastricht, Maastricht: Encarna Gomez-Garcia, Rien Blok.

Epidemiological study of BRCA1 & BRCA2 mutation carriers (EMBRACE): DE is the PI of the study. EMBRACE Collaborating Centers are: Coordinating Centre, Cambridge: Susan Peock, Margaret Cook, Clare Oliver, Debra Frost. North of Scotland Regional Genetics Service, Aberdeen: Zosia Miedzybrodzka, Helen Gregory. Northern Ireland Regional Genetics Service, Belfast: Patrick Morrison. West Midlands Regional Clinical Genetics Service, Birmingham: Trevor Cole, Carole McKeown, Lucy Burgess. South West Regional Genetics Service, Bristol: Alan Donaldson. East Anglian Regional Genetics Service, Cambridge: Joan Paterson. Medical Genetics Services for Wales, Cardiff: Alexandra Murray, Mark Rogers, Emma McCann. St James’s Hospital, Dublin & National Centre for Medical Genetics, Dublin: John Kennedy, David Barton. South East of Scotland Regional Genetics Service, Edinburgh: Mary Porteous. Peninsula Clinical Genetics Service. Exeter: Carole Brewer, Emma Kivuva, Anne Searle, Selina Goodman. West of Scotland Regional Genetics Service, Glasgow: Rosemarie Davidson, Victoria Murday, Nicola Bradshaw, Catherine Watt, Lesley Snadden, Mark Longmuir. South East Thames Regional Genetics Service, Guys Hospital London: Louise Izatt, Gabriella Pichert, Caroline Langman. North West Thames Regional Genetics Service. Harrow: Huw Dorkins. Leicestershire Clinical Genetics Service, Leicester: Julian Barwell. Yorkshire Regional Genetics Service, Leeds: Tim Bishop, Carol Chu, Julie Miller. Merseyside & Cheshire Clinical Genetics Service. Liverpool: Ian Ellis. Manchester Regional Genetics Service, Manchester: D Gareth Evans, Fiona Lalloo, Felicity Holt. North East Thames Regional Genetics Service, NE Thames: Alison Male, Anne Robinson. Nottingham Centre for Medical Genetics, Nottingham: Carol Gardiner. Northern Clinical Genetics Service, Newcastle: Fiona Douglas, John Burn. Oxford Regional Genetics Service, Oxford: Lucy Side, Lisa Walker, Sarah Durell. The Institute of Cancer Research and Royal Marsden NHS Foundation Trust: Ros Eeles, Susan Shanley, Nazneen Rahman, Richard Houlston, Elizabeth Bancroft, Lucia D’Mello, Elizabeth Page, Audrey Ardern-Jones, Anita Mitra. North Trent Clinical Genetics Service, Sheffield: Jackie Cook, Oliver Quarrell, Cathryn Bardsley. South West Thames Regional Genetics Service, London: Shirley Hodgson.

The GEMO study (Genetic Modifiers of cancer risk in *BRCA1/2* mutation carriers: Cancer Genetics Network “Groupe Génétique et Cancer”, Fédération Nationale des Centres de Lutte Contre le Cancer, France) Collaborating Centers: Coordinating Centres, Unité Mixte de Génétique Constitutionnelle des Cancers Fréquents, Hospices Civils de Lyon / Centre Léon Bérard and UMR5201 CNRS, Université de Lyon, Lyon: Olga Sinilnikova, Laure Barjhoux, Sophie Giraud, Mélanie Léone, Sylvie Mazoyer; and INSERM U509, Service de Génétique Oncologique, Institut Curie, Paris: Dominique Stoppa-Lyonnet, Marion Gauthier-Villars, Claude Houdayer, Virginie Moncoutier, Muriel Belotti, Antoine de Pauw. Institut Gustave Roussy, Villejuif: Brigitte Bressac-de-Paillerets, Audrey Remenieras, Véronique Byrde, Corinne Capoulade, Gilbert Lenoir. Centre Jean Perrin, Clermont–Ferrand: Yves-Jean Bignon, Nancy Uhrhammer. Centre Léon Bérard, Lyon: Christine Lasset, Valérie Bonadona. Centre François Baclesse, Caen: Agnès Hardouin, Pascaline Berthet. Institut Paoli Calmettes, Marseille: Hagay Sobol, Violaine Bourdon, François Eisinger. Groupe Hospitalier Pitié-Salpétrière, Paris: Florence Coulet, Chrystelle Colas, Florent Soubrier. CHU de Arnaud-de-Villeneuve, Montpellier: Isabelle Coupier. Centre Oscar Lambret, Lille: Jean-Philippe Peyrat, Joëlle Fournier, Philippe Vennin, Claude Adenis. Centre René Huguenin, St Cloud: Etienne Rouleau, Rosette Lidereau, Liliane Demange, Catherine Nogues. Centre Paul Strauss, strasbourg: Danièle Muller, Jean-Pierre Fricker. Institut Bergonié, Bordeaux: Michel Longy, Nicolas Sevenet. Institut Claudius Regaud, toulouse: Christine Toulas, Rosine Guimbaud, Laurence Gladieff, Viviane Feillel. CHU de Grenoble: Dominique Leroux, Hélène Dreyfus, Christine Rebischung. CHU de Dijon: Laurence Olivier-Faivre. CHU de St-Etienne: Fabienne Prieur. Centre Antoine Lacassagne, Nice: Marc Frénay. Creighton University, Omaha, USA: Henry T. Lynch.

The following are GC-HBOC collaborating centers: Institute of Human Genetics, Charite-University Medical Centre, Berlin, Germany; Institute of Human Genetics, University of Leipzig, Leipzig, Germany; Department of Gynaecology and Obstetrics, Technical University, Dresden, Germany; Department of Obstetrics and Gynaecology, Technical University, Munich, Germany; Department of Obstetrics and Gynaecology, University of Ulm, Germany; Institute of Human Genetics, University of Heidelberg, Germany; Institute of Human Genetics, University of Frankfurt, Frankfurt, Germany; Department of Obstetrics and Gynaecology, Division of Molecular Gynaeco-Oncology, University of Cologne, Germany; Molecular Genetics Laboratory, Department of Obstetrics and Gynaecology, University of Duesseldorf, Germany; Institute of Human Genetics, University of Münster, Münster, Germany; Department of Obstetrics and Gynaecology, University of Schleswig-Holstein, Campus Kiel, Germany; Institute of Cellular and Molecular Pathology, Medical University, Hannover, Germany; Institute for Medical Informatics, Statistics and Epidemiology, University of Leipzig, Germany.

Milan Breast Cancer Study Group (MBCSG)

Spanish National Cancer Centre (CNIO)

HEBCS Helsinki Breast Cancer Study
